# Supplementary figures and images for: The retinoid X receptor has a critical role in synthetic rexinoid-induced increase in cellular all-trans-retinoic acid
Source: PLoS One. 2024 Apr 1;19(4):e0301447. doi: 10.1371/journal.pone.0301447 (PMC10984533; doi:10.1371/journal.pone.0301447)

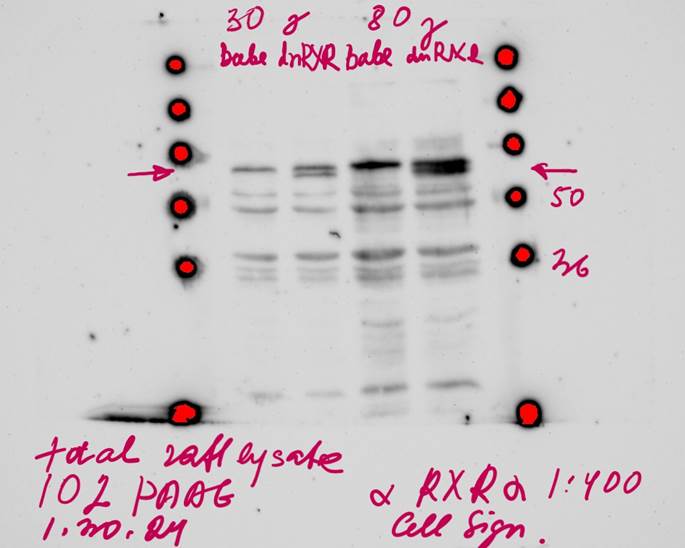

Supplement: S1 Fig — (JPG) [file pone.0301447.s001.jpg]

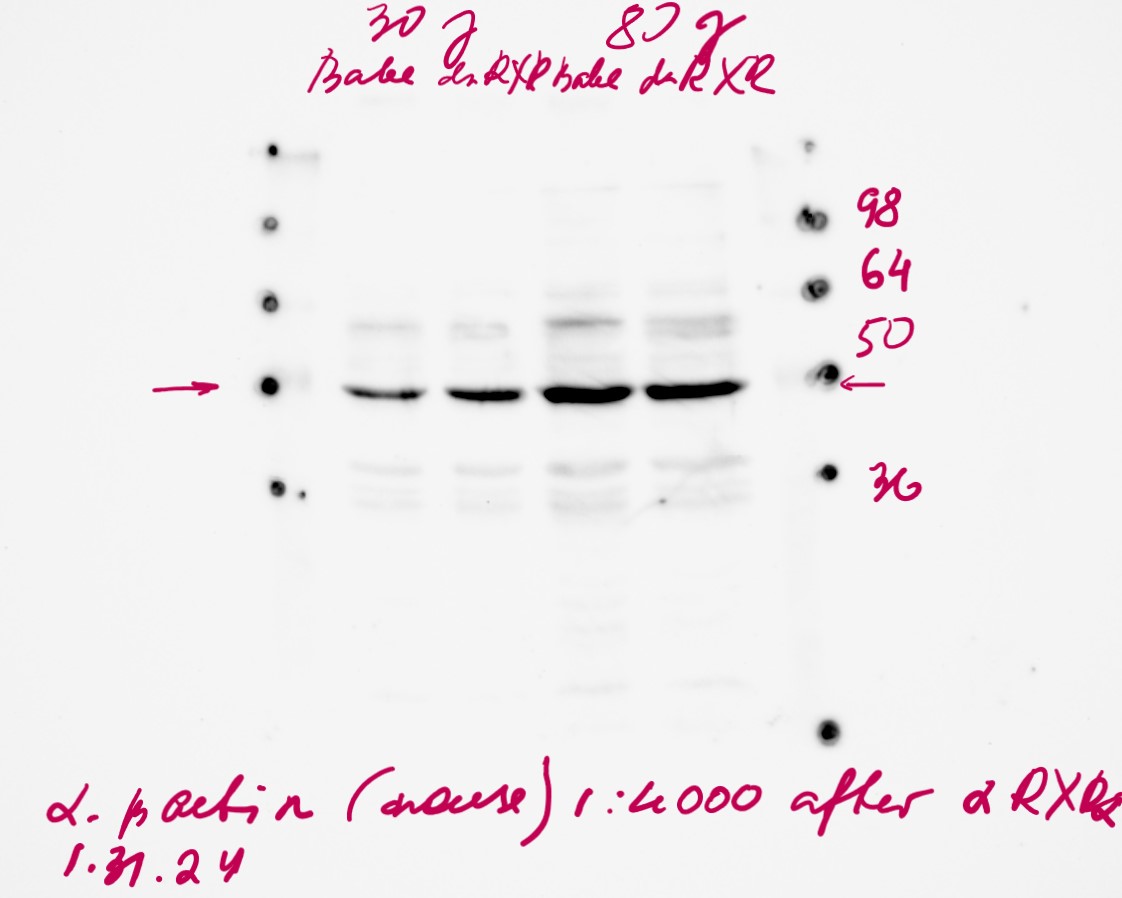

Supplement: S2 Fig — (JPG) [file pone.0301447.s002.jpg]

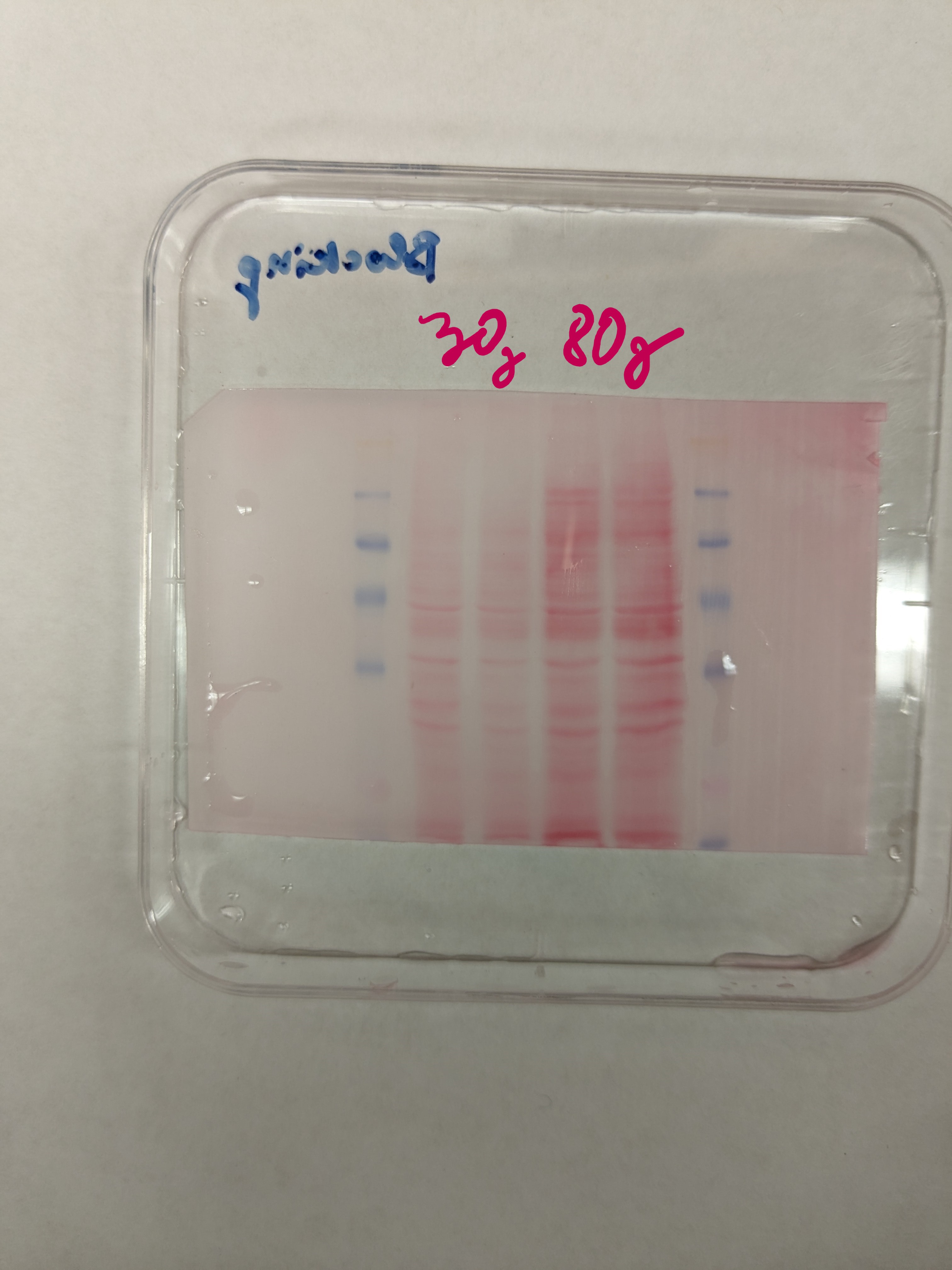

Supplement: S3 Fig — (JPG) [file pone.0301447.s003.jpg]
