# Supplementary material for: The retinoid X receptor has a critical role in synthetic rexinoid-induced increase in cellular all-trans-retinoic acid
Source: PLoS One. 2024 Apr 1;19(4):e0301447. doi: 10.1371/journal.pone.0301447 (PMC10984533; doi:10.1371/journal.pone.0301447)
Supplement: Attachment — Submitted filename: Response to Reviewers.pdf [file pone.0301447.s014.pdf]

We appreciate the reviewers' comments and comprehensively addressed the points raised during the review process. Below is our point-by-point response to the comments.

Reviewer #1:
1) Organotypic skin raft culture:

i) *Are donor foreskins from a single individual used to generate cultures for each replicate of an experiment, or was pooled donor tissue used to generate cultures? This information would be helpful to the understanding of possible biological/individual variability that this model is subject to.*

A typical experiment described in this manuscript requires up to 30 epidermis raft cultures to cover all the treatment variants and controls. As primary keratinocytes have limited proliferation capacity before becoming senescent, the number of keratinocytes obtained from the individual foreskin sample is not sufficient to seed that many cultures. Typically, PHKs from 2-3 individual foreskin samples are pooled at the time of PHK isolation, and these PHKs are frozen as a single batch of cells. For each experiment, PHKs are expanded and rafts are prepared from a single batch of cells, albeit originally derived from 2-3 pooled foreskin samples. Thus, within the experiment all the control and treated samples have the same origin (i.e., the whole set of epidermis samples used for RNAseq was cultured simultaneously from the same starting material).

We do indeed observe some variability in the strength of the response to rexinoid treatment between sets of epidermis cultures prepared from different pools of PHKs. It can be manifested as more or less profound changes in histology, but we have never observed a change in the direction of the response, or absence of the response to the rexinoid treatment. For example, the histology sections shown in Fig. 2 were prepared from the set of epidermis rafts cultured simultaneously from a single pool of PHKs. However, the changes observed here were observed in two additional experiments, where epidermal rafts were prepared from the different pools of primary keratinocytes.

ii) *Is the skin raft model maintained in media that have certain critical reagents (e.g. fetal bovine serum) known to contain retinoids and other hormones? If so, the authors may need to carefully consider the potential contributions and confounding effects of certain components in the serum.*

Yes, the FBS typically contains some amount of retinol, but the same medium is used for the control samples as well as for treated samples to account for the variation between batches of FBS.

2) *RNA seq studies: What was considered a biological replicate and how many biological replicates were performed?*

Individual epidermis culture was considered a biological replicate; each treatment group had 4 individual skin raft cultures. The effects of rexinoids reported here are similar to those obtained with different batches of keratinocytes as reported in our previous manuscripts.

3) *Given the altered RA concentrations, it would be useful to discuss whether the expression of classically known RA synthesis and/or catabolism genes was affected significantly?*

Some of these genes were included in qPCR analysis in Fig 3 (*STRA6, LRAT, DHRS3, RBP1, SDR16C5*). Analysis of the same genes and others (CYP26B1, RDH10) in the same organotypic epidermis system has been described earlier in Wu et al. (PLoS One. 2016;11(4):e0153556) and Melo et al. (J Biol Chem. 2023;299(1):102746).

Reviewer #2: Tracking #: PONE-D-23-37860 – Belyaeva et al.

Reviewer 2 expressed concern regarding our H12 deletion mutant that was included in our analysis and the interpretation of our results. Specifically, the reviewer stated the following:

"Although the authors' choice of a receptor that loses the ability to be activated by a rexinoid agonist seems relevant for assessing RXR's contribution to rexinoid-mediated effects, H12 deletion proves too drastic for the purposes of this study. This mutant was insufficiently controlled by the authors. This makes for a paradoxical work with very uneven conclusions and interpretations of values. Indeed, it has been shown that such a deletion induces the ability of RXR to interact with the transcriptional corepressors NCoR and SMRT; wild-type RXR does not interact with these corepressors (Hu and Lazar, 1999, Nature 402:93-6). This results in dnRXR-mediated transcriptional repression, even in the context of heterodimers containing dnRXR (and thus the one formed with RAR). So the effects observed in the presence of this RXR mutant are probably due to induced repression rather than loss of activation. The interpretation of the results obtained needs to be re-evaluated. Not only does the assertion that an endogenous rexinoid ligand exists and is active (see pages 11 and 14) need to be qualified, but also the transcriptomics analyses relating to dnRXR need to be reconsidered."

The 1999 paper by Hu and Lazar originally identified that the deletion of H12 results in the increased ability of RXR to interact with transcriptional corepressors NCoR and SMRT. This work was done on an RXR/Thyroid Receptor (TR) complex and not RXR:RAR heterodimers. Additionally, the reviewer's statement that "wild-type RXR does not interact with these corepressors" is not supported by subsequent studies that indicate that RXR:RAR heterodimers constitutively exist in a repressed state that includes bound corepressors NCoR and SMRT (Love JD, Gooch JT, Benko S, Li C, Nagy L, Chatterjee VK, Evans RM, Schwabe JW. The structural basis for the specificity of retinoid-X receptor-selective agonists: new insights into the role of helix H12. J Biol Chem. 2002 Mar 29;277(13):11385-91. doi: 10.1074/jbc.M110869200; Benko S, Love JD, Beládi M, Schwabe JW, Nagy L. Molecular determinants of the balance between co-repressor and co-activator recruitment to the

retinoic acid receptor. J Biol Chem. 2003 Oct 31;278(44):43797-806. doi: 10.1074/jbc.M306199200. Kao HY, Han CC, Komar AA, Evans RM. Co-repressor release but not ligand binding is a prerequisite for transcription activation by human retinoid acid receptor alpha ligand-binding domain. J Biol Chem. 2003 Feb 28;278(9):7366-73. doi: 10.1074/jbc.M207569200).

In 2019, Cordeiro et al. reported on a detailed structural study that looked at the binding of NCoR to the RXR/RAR heterodimer and concluded that NCoR binding is multivalent with interactions with both RAR and RXR. NCoR has a higher affinity for RAR than RXR (Cordeiro et al. Structure, 2019). They also demonstrated that the deletion of RXR helix 12 increases the affinity of NCoR. They hypothesized that the presence of the highly dynamic H12 acts as a screen to limit NCoR binding. They also reported that the interaction of NCoR with the RXR/RAR heterodimer was reduced in the presence of the RAR agonist TTNPB. However, when the agonist was added to an RXRdeltaH12/RAR heterodimer the affinity for NCoR was not reduced, indicating the loss of RXR helix 12 increased the affinity for NCoR. In their discussion they wrote:

"In the absence of ligand, there exists equilibrium between a major population of asymmetric binding of N-CoRNID to RXR/RAR and a minor population of doubly bound N-CoRNID (deck-binding mode) in which both CoRNR boxes simultaneously interact with the heterodimer, accounting for the cooperativity of the interaction. The major species of the asymmetric binding mode can be reasonably assigned to N-CoRNID binding to RAR through the CoRNR1 motif, which presents the strongest local affinity."

Based on Cordeiro et al's in vitro structural analysis we would have presumably seen a significantly repressed state in our ex vivo skin raft system with the presence of a deltaH12dnRXRα. <u>However, this is not the case</u>. ATRA levels are slightly diminished in the presence of the dnRXRα compared to empty vector but they are not abolished (Fig 4). There is still a steady state level of ATRA measured. Figure 1C demonstrates that only *MUC21* out of 9 known genes to be regulated by ATRA is diminished. Additionally, the histology of the raft cultures shows no dramatic changes in the morphology and stratification of skin rafts in the presence of dnRXRα which is consistent with a small decrease in ATRA signaling. If there was a major decrease in ATRA levels, the keratinocytes would not proliferate and form stratified layers.

Significantly, the addition of UAB30 or UAB110 agonists did not increase ATRA levels in the presence of dnRXRα. They are not decreased either. The reviewer suggests that the increased affinity of corepressors such as SMRT and NCoR for RXR without H12 would explain these effects. However, our gene profiling results show that the impact of dnRXRα is gene-specific, e.g., UAB110-induced upregulation of *MUC21* and *LRAT* is not abolished by dnRXRα. The upregulation of other ATRA-sensitive genes by rexinoids is diminished in the presence of dnRXRα, <u>but there is still a slight gradient effect when going from the moderate</u>

agonist UAB30 to the strong agonist UAB110 for genes such as *FLG* and *SDR16C5*. For other genes there is minimal difference between the two rexinoids (*GABRP, STRA6, RARB, DHRS3*), but the overall effect is not repression.

One possibility that is not addressed by Cordeiro et al. is the nature of NCoR binding in the presence of an RXR agonist as we are using in our experiments. We have demonstrated in our previous publications that addition of rexinoids (UAB30 and UAB110) in our skin raft cultures potentiates the transcriptional activity of pre-existing resident ATRA and that there is an overall upregulation of ATRA synthesis genes. Others have demonstrated that agonist can still bind RXR in the absence of H12 (Liu et al., JBC 2004; Mascrez et al, Development, 1998). In the presence of agonist bound to RXRdeltaH12, would NCoR still be able to interact with RXR lacking H12 with the same affinity? Agonist binding to RXR does not exclusively reorder H12 but also the entire RXR LBD to create a ligand binding pocket that significantly changes the positions of H3 and H4. This possibility was not addressed by Cordeiro et al in their structural studies. So, from the Cordeiro et al studies it is unknown whether rexinoid binding (endogenous or otherwise) by dnRXRalpha without helix 12 would release corepressor binding. Our data in the raft cultures suggests that there is some level of endogenous ligand regulation occurring.

Whether the mild effect of potential NCoR binding to RXRdeltaH12 on gene expression reflects the differences between an in vitro assay system and an organ system is not clear at this point. Perhaps, the binding of endogenous RXR ligand in keratinocytes displaces the NCoR.

Nevertheless, taking into account these uncertainties, we toned down our conclusion about the existence of endogenous ligand as reflected in the revised manuscript.

1/ The choice of the H12-deleted RXR mutant is debatable. Other options may be considered. Hu and Lazar's paper shows that certain mutations in the hydrophobic groove of RXR (in H3 and H4, the domain mentioned by the authors on page 13) inhibit activation by preventing the recruitment of coactivators, while not allowing the binding of corepressors. Another possibility would be to use mutants of the RXR ligand-binding pocket that prevent rexinoid binding (Le Maire et al., 2022, JME, 69:377-390).

Thank you for the references and suggesting alternative mutants that could be employed in our future research. Our goal for this study was to test whether a functional RXR is essential for mediating the effects of rexinoids. If the side effect of H12-deleted RXR mutant is increased binding of NCoR, that only strengthens our conclusion that intact RXR is necessary for observing the biological effects of UAB30 and UAB110.

Also, if it is possible to use siRNAs in the model exploited in this study, siRNAs directed against RXRalpha, but also RARgamma, would address the questions posed by the authors.

Thank you for this suggestion, unfortunately, it is not technically possible to use siRNAs in our model.

2/ Gene expression analyses by qPCR (Fig 1 and 3) use only 3 replicates. This is perhaps insufficient for clear conclusions. For example for the effect of dnRXR on the expression of MUC21 (Fig 1C) a large variation is observed for the control condition which leads the authors to consider a significant difference between the control and the dnRXR condition, and this only for MUC21. This test should be repeated to confirm the interpretation made. In a more nuanced way, it seems to me that the expression of dnRXR causes a slight decrease in the expression of several of the genes studied.

Just to clarify, Fig. 1C shows a fold-change in expression of several ATRA-regulated genes upon overexpression of dnRXRα (dnRXRα-raft) or of empty vector (EV-raft) in comparison to control (untransfected) rafts. The values of controls are not in the plot.

We do observe a greater variation in the expression of those genes that are ***highly sensitive to ATRA levels***, such as *MUC21* and *STRA6*, after treatment with UAB series rexinoids. These same genes also showed higher variability in our previous reports Wu et al. (PLoS One. 2016;11(4):e0153556) and Melo et al. (J Biol Chem. 2023;299(1):102746).

3/ Although the reported results indicate an effect of dnRXR, and thus that its expression is effective, it is difficult to evaluate the relative expressions of endogenous wild-type and exogenous mutated forms of RXRalpha. The use of a tagged dnRXR would make it possible to differentiate the two proteins. Also the Western blot conditions could be optimized to improve the separation even if the molecular mass difference is small.

The Western blot with improved separation of bands is now shown in Fig. 1B. 10% PAAG instead of 12% PAAG and lower protein load (30 μg) allowed for better separation of the recombinant AF2 truncated mutant and the native RXRα expressed in the organotypic epidermis.

4/ For transcriptomics analyses, authors often refer to retinoid target genes. Are there data in this human epidermis model for retinoic acid? Such data would be valuable for being able to precisely correlate the effects of rexinoids to genes and pathways regulated by retinoic acid.

Yes, there are numerous genes expressed in skin raft cultures that are regulated by retinoic acid: *COL1A1* [33], *CXCL14* [34], *KRT2* [35], *KLK13* [36], *CCN1* [37], *SCD* [38], *S100P* [39], *EGR1* [40], etc.

5/ It would be nice to have the structure of the UAB30 and UAB110 rexinoids, as well as their corresponding chemical formula.

Please see the structures in Atigadda VR, Xia G, Deshpande A, Wu L, Kedishvili N, Smith CD, Krontiras H, Bland KI, Grubbs CJ, Brouillette WJ, Muccio DD. Conformationally Defined Rexinoids and Their Efficacy in the Prevention of Mammary Cancers. J Med Chem. 2015 Oct 8;58(19):7763-74. doi: 10.1021/acs.jmedchem.5b00829.

6/ Typo: page 13 line 7, "overexpression of dnRXRa" instead of "RXRa".

Thank you, this has been corrected.
